# Baseline, Impact and Surveillance Trachoma Prevalence Surveys in Burundi, 2018–2021

**DOI:** 10.1080/09286586.2023.2213776

**Published:** 2023-07-03

**Authors:** Victor Bucumi, Elvis Muhimpundu, Amadou Alfa Bio Issifou, Stephanie Akweyu, Nick Burn, Johan Willems, Junénal Niyongabo, Aba Elvis, Gamael Koizan, Anna Harte, Sarah Boyd, Rebecca Willis, Ana Bakhtiari, Cristina Jimenez, Clara Burgert-Brucker, KHM Martin Kollmann, Anthony W. Solomon, Emma M. Harding-Esch, Rose Marie Gashikanyi

**Affiliations:** a Département En Charge des Maladies Tropicales, National Integrated Programme for the Control of Neglected Tropical Diseases and Blindness (PNIMTNC), Bujumbura, Burundi; b Department of Ophthalmology, University of Parakou, Parakou, Benin; c Inclusive Eye Health and Neglected Tropical Diseases Initiative, CBM international; d Programme National de la Santé Oculaire et de la lutte contre l’Onchocercose, Côte d’Ivoire; e Ministère de la Santé et de l’Hygiène Publique, Cote d’Ivoire; f Clinical Research Department, London School of Hygiene & Tropical Medicine, London, UK; g International Trachoma Initiative, Task Force for Global Health, Atlanta, Georgia, USA; h Sightsavers International, Haywards Heath, UK; i Global Health Division, RTI International, Atlanta, Georgia, USA; j Department of Control of Neglected Tropical Diseases, World Health Organization, Geneva, Switzerland

**Keywords:** Burundi, prevalence, trachoma, trichiasis, Tropical Data, WASH

## Abstract

**Purpose:**

Trachoma is an eye disease caused by the bacterium Chlamydia trachomatis (Ct). It can lead to permanent vision loss. Since 2007, Burundi has included trachoma elimination as part of its fight against neglected tropical diseases and blindness. This study presents the results of trachoma baseline, impact and surveillance surveys conducted in Burundi between 2018 and 2021.

**Methods:**

Areas were grouped into evaluation units (EU) with resident populations of between 100,000 and 250,000 people. Baseline surveys were conducted in 15 EUs, impact surveys in 2 EUs and surveillance surveys in 5 EUs; in each survey, 23 clusters of about 30 households were included. Consenting residents of those households were screened for clinical signs of trachoma. Access to water, sanitation and hygiene (WASH) was recorded.

**Results:**

A total of 63,800 individuals were examined. The prevalence of TF in 1–9-year-olds was above the elimination threshold of 5% in a single EU at baseline, but fell below the threshold in subsequent impact and surveillance surveys. The prevalence of TT was below the 0.2% elimination threshold in ≥15-year-olds in all EUs surveyed. A high proportion (83%) of households had access to safe drinking water, while only a minority (~8%) had access to improved latrines.

**Conclusion:**

Burundi has demonstrated the prevalence levels necessary for trachoma elimination status. With continued effort and the maintenance of existing management plans, trachoma elimination in Burundi is within reach.

## Introduction

Trachoma is an eye disease caused by the bacterium *Chlamydia trachomatis* (Ct).^1^ It is mainly found in areas where access to health care, water and sanitation is inadequate. The main clinical sign of trachoma in children is inflammation of the tarsal conjunctiva, with additional signs (as described in the World Health Organization’s (WHO) simplified trachoma grading system) indicative of the scarring complications of that inflammation.^2^ In brief, the simplified system has five signs: trachomatous inflammation – follicular (TF), trachomatous inflammation – intense (TI), trachomatous scarring (TS), trachomatous trichiasis (TT) and corneal opacity (CO). TF and TI are signs of active trachoma and can be treated with antibiotics such as azithromycin.^3^ Pfizer donates azithromycin (Zithromax®, USA) to trachoma-endemic countries through the International Trachoma Initiative.

Trachoma’s elimination as a public health problem has been a WHO-supported target since 1996, when the WHO Alliance for the Global Elimination of Trachoma by 2020 (GET2020) was launched.^4^ A key focus of the Alliance has been supporting implementation of the SAFE strategy in countries in which trachoma is endemic. The SAFE strategy has four components: Surgery for TT, antibiotics to clear ocular Ct infection, and facial cleanliness and environmental improvement, particularly improved access to water and sanitation, to limit ocular Ct transmission.^5^ Population-based surveys are critical at baseline, and subsequently, to periodically estimate the prevalence of TF and TT in order to guide decision-making on the need for SAFE interventions.^6,7^

Surveys use established survey methods that incorporate epidemiologically appropriate sample sizes and sampling patterns, certified field teams, agreed water, sanitation and hygiene (WASH) questionnaires, electronic data capture and a full suite of quality assurance and quality control measures.^8–10^ Countries are separated into evaluation units (EUs), which are administrative units for healthcare management and are generally equivalent to a health district, with a population of between 100,000 to 250,000 people.^11^ An accurate assessment of the burden of disease is essential to prioritize the use of limited resources, plan services for those who need them, prevent disease complications and progress towards the elimination target.

According to WHO guidance,^12^ surveys that reveal EUs to have a prevalence of TF ≥ 5% in 1–9-year-olds should implement the A, F and E components of the SAFE strategy, with the number of planned antibiotic mass drug administration (MDA) rounds dependent on the TF prevalence category. Six to twelve months after the last planned MDA is completed, WHO recommends impact surveys to determine whether MDA should be continued or can be stopped. EUs in which the TF prevalence is <5% should be subject to a surveillance survey, done at least two years after the impact survey^12^ to determine if TF prevalence has remained below the elimination threshold after cessation of MDA. Surveys that reveal a TT burden at or above the 0.2% threshold are recommended to implement the “S” part of the SAFE strategy, which involves improving access to and acceptance of trichiasis surgery and epilation.^13^ Areas with TT prevalence at or above the threshold, but TF prevalence below the 5% threshold, are recommended for additional TT-only surveys to increase the precision of the TT estimates in affected EUs.^14,15^

In 2007, the Burundi Ministry of Health included trachoma in its Neglected Tropical Diseases (NTDs) Control Programme fieldwork, which was conducted nationwide to estimate the prevalence of schistosomiasis, soil-transmitted helminths and lymphatic filariasis in the country’s 45 health districts. A single sentinel site was surveyed for trachoma in each health district, where a rapid assessment of 50 adults and 50 children were examined.^16^ Active trachoma was found at all sites. Burundi therefore included trachoma elimination as part of its integrated programme for control of NTDs and blindness (Ministerial Order No. 630/158/1/210). Between 2009 and 2010, TF prevalence surveys of twenty-three districts confirmed that 12 districts were endemic. Four of these had a TF prevalence >10% and received one round of Zithromax treatment.

The 2009–2010 baseline surveys were conducted before standardized methodologies were made available by the Global Trachoma Mapping Project; they did not record data on TT, were somewhat underpowered for estimating TF prevalence with appropriate accuracy,^9^ and some EUs experienced issues with unreliable sampling due to community mistrust of random selection and household visits (Table 1). In spite of this, in 2011, Burundi implemented antibiotic MDA using azithromycin and tetracycline in the three districts with TF prevalence ≥10% (Buhiga, Nyabikere, and Muyinga)^16,17^ and, in 2012, in the district of Rutana. The 19 EUs with TF prevalence <10% in the 2009–2010 surveys were not treated.

**Table 1.** Trachomatous inflammation – follicular (TF) prevalence in 1–9-year-olds in the 23 districts originally surveyed in 2009–2010; antibiotic mass drug administration (MDA) year; and the year of and TF prevalence (in 1–9-year-olds) from baseline, impact and surveillance surveys for the subsequent Tropical Data-supported surveys in Burundi in 2018–2021. EU; Evaluation Unit.

EU/district	TF prevalence in 1–9-year-olds, 2009–2010	Antibiotic MDA year	New baseline survey year (TF prevalence)	Impact survey year (TF prevalence)	Surveillance survey year (TF prevalence)
Muyinga	13.6	2011	-	2012* (3.47)	2020 (0.82)
Buhiga	13.1	2011	-	2012* (1.60)	2020 (0.38)
Nyabikere	12.5	2011	-	2012* (1.50)	2020 (0.20)
Rutana	11.9^†^	2012	-	2018 (2.34)	2020 (0.52)
Gashoho	9.4	2019	2018 (7.16)	2019 (3.48)	2021 (0.33)
Cankuzo	3.2	-	2021 (0.10)	-	-
Mukenke	3.3	-	2020 (0.22)	-	-
Makamba	2.7	-	2021 (0.32)	-	-
Nyanza-lac	0.9	-	2021 (0.00)	-	-
Busoni	4.3	-	2018 (2.44)	-	-
Butezi	6.8 ^†^	-	2018 (4.17)	-	-
Gihofi	8.7^†^	-	2018 (1.24)	-	-
Giteranyi	8.9	-	2018 (4.65)	-	-
Kirundo	4.3	-	2018 (1.39)	-	-
Gahombo	5.6	-	2018 (4.22)	-	-
Mutaho	6	-	2018 (1.82)	-	-
Kinyinya	7.6	-	2018 (1.15)	-	-
Musema	3.5	-	2018 (3.53)	-	-
Kiremba	8.6	-	2018 (3.97)	-	-
Murore	3.4^†^	-	-	-	-
Ruyigi	2.3^†^	-	-	-	-
Rumonge	1.5	-	-	-	-
Vumbi	3.3	-	-	-	-

Note: *surveys conducted prior to Tropical Data.

^†^estimates based on unreliable sampling (please see text).

In 2018, it was considered advisable to conduct new baseline surveys to re-evaluate the situation and plan further actions. The first Burundi EUs to be re-surveyed using the standardized techniques were those that had had TF prevalence estimates of 5.0–9.9% in 2009–2010 and unreliable sampling (Table 1). During later surveys in 2020–2021, EUs with TF prevalence estimates below 5% in 2009–2010 (Cankuzo, Makamba, Mukenke and Nyanza-Lac) were re-surveyed to re-check baseline prevalence estimates. These surveys provide important information on the state of trachoma in Burundi and will help target resources where needed.

This paper presents the results of new baseline, impact and surveillance surveys conducted across Burundi from 2018–2021.

## Method

### Study ethics

Protocols were approved by the Burundi National Ethics Committee (February 2018) and the research ethics committee of the London School of Hygiene & Tropical Medicine (reference: 16105). Residents of selected households were informed of the purpose and procedures of the survey in Kirundi, the local language, before being asked to give verbal consent; parents or guardians gave consent for participation of children and could terminate the child’s participation at any time without giving a reason. Any participant found to have active trachoma received 1% tetracycline ointment, or azithromycin syrup or tablets, free of charge, and participants with TT were offered free surgery.

### Study design

Three types of trachoma prevalence surveys were used: baseline, impact and surveillance, with all three using a common methodology.^8,9^ Areas were divided into EUs of 100,000 to 250,000 people, with populations estimated using the general census of population and housing conducted in 2008.^18^ In total, 22 surveys were conducted in 19 EUs, including 15 baseline, 2 impact and 5 surveillance surveys.

The primary outcome measure was TF prevalence in 1–9-year-olds, with TT prevalence data collected in people ≥15 years a secondary outcome. The target number of children to be examined per EU was 1222 for baseline and impact surveys conducted in 2018, following the methodology of the Global Trachoma Mapping Project (GTMP),^8^ and 1164 for all later surveys, following the updated WHO methodology.^10^ In 2018, sample size was calculated using predicted prevalence 10%, margin of error 3%, design effect 2.65,^19^ standard deviation corresponding to 95% confidence intervals (1.96) and an inflation factor of 1.2 to account for non-response. In later surveys, the predicted prevalence was 4%, margin of error 2%, and the design effect 2.63.^10,20^ A two-stage cluster sampling approach was used in each EU at each timepoint; first-stage clusters were villages, chosen systematically with probability of selection proportional to population size from the list of all villages in the EU. The number of first-stage clusters to be surveyed was calculated based on the number of households a team could consistently survey in one day (30) and the estimated 1.86 children aged 1–9 years per household; this resulted in a required number of 23 clusters per EU. Anyone one year of age or above who had been living in the household for at least one month was eligible for inclusion.

### Examination

Graders and recorders were trained in accordance with the Tropical Data training system,^21,22^ which uses the principles of the GTMP.^8,9^ In brief, all graders attended a 2–3 day training workshop on the WHO simplified trachoma grading system with slide training on the various clinical signs of trachoma. Due to the lack of trachoma cases, the first training of graders in 2017 was carried out in Ethiopia. Trainees used practice slide sets and underwent field-based intergrader agreement (IGA) tests for TF, with a required kappa score of ≥0.7 in order to achieve certification. Recorders were trained over 2–3 days to correctly fill out the hard copy data collection form and input the information into the Tropical Data app. Recorders underwent a recorder reliability test with a required pass rate of 90%. The training ended with team training so the graders and recorders could practice working together. All consenting eligible residents were screened by a doctor or nurse certified as a trachoma grader by Tropical Data. The upper and lower eyelids were examined for signs of trichiasis. The upper eyelids of both eyes were everted to reveal the tarsal conjunctiva and examined using a 2.5× magnification loupe, and a flashlight if necessary. WHO’s simplified trachoma grading system^2,23^ was used. In surveys from 2019 onwards, follicle size guides were used to aid the accurate diagnosis of TF.^24^

### Definitions

During the course of the surveys described here, two different definitions of TT were used. Surveys conducted before 2019 defined TT as the presence of one or more eyelashes from the upper or lower eyelid touching the eyeball. In surveys using this definition, field teams did not distinguish between upper and lower eyelids when recording cases of TT. However, diseases other than trachoma can cause eyelashes from the lower eyelids to invert,^23^ and in 2018 the 4th Global Scientific Meeting on Trachoma (GSM4)^15^ recommended that only inverted eyelashes of the upper eyelid should be considered when evaluating *trachomatous* trichiasis. As a result, since 2019, the WHO simplified trachoma grading system^23^ has defined TT as the presence of at least one eyelash from the upper eyelid touching the eyeball, or evidence of recent epilation of in-turned eyelashes from the upper eyelid; trichiasis is defined as the presence of at least one eyelash from the upper or lower eyelid touching the eyeball. Surveys implemented from 2019 onwards recorded from which eyelid in-turned eyelashes came from, and if there was any associated TS. TF is defined as the presence of five or more follicles, each at least 0.5 mm in diameter, in the central part of the upper tarsal conjunctiva. TS is defined as the presence of easily visible conjunctival scarring, seen as white lines, bands or sheets in the upper tarsal conjunctiva.

### Access to water, sanitation and hygiene

Field teams asked the head of the household for information on WASH access, including access to drinking and washing water, type of water source, type of latrine and proximity. Drinking water and washing water were classified as surface, unimproved or improved based on source and type, as defined by the WHO Joint Monitoring Programme^25^; water came from an improved source if purchased or piped, while rainwater and unprotected dug wells were classified as unimproved. The type of latrine was classified as improved if it was a solid and/or plumbed structure, and not improved if open drains, pits, buckets or hanging toilets were used or open defecation was practised. It should be noted that prior to 2019, the latrine type “Flush/pour flush to open drains” counted as “improved”.

### Data analysis

Estimates of the prevalence of TF and TT were calculated using previously described methods.^8^ In brief, EU-level TF prevalence was derived as the mean of the age-adjusted first-stage cluster-level proportions; and EU-level TT prevalence as the mean of the age- and gender-adjusted first-stage cluster-level proportions. A bootstrapping method was used to calculate 95% confidence intervals around the prevalence estimates.^26^ Mixed-effect linear models were constructed to investigate the relationship between the presence or absence of TF and WASH variables, using the R glmer package.^27^ The random effects tested in the model included EU, cluster and household. Models including all fixed effects and combinations of random effects were tested to determine the best fit using Akaike’s information criteria.^28^

## Results

**Figure 1.**
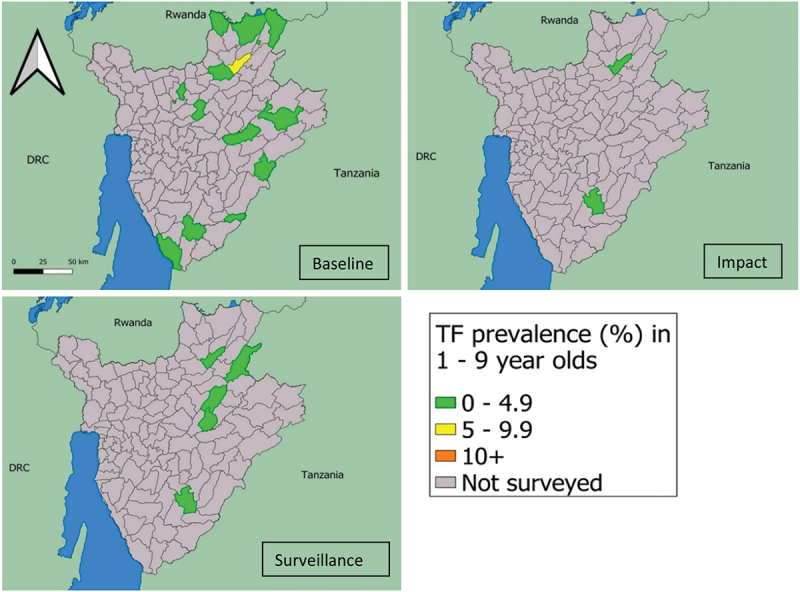
Prevalence of trachomatous inflammation – follicular (TF), Burundi trachoma prevalence surveys, 2018–2021, by survey type: (a) Baseline, (b) Impact and (c) Surveillance. The boundaries and names indicated and the designations used on this map do not imply the expression of any opinion of any kind on the part of the authors, or the institutions with which they are affiliated, concerning the legal status of a country, territory, city or region or its authorities, or concerning the delimitation of its borders.

A total of 63,800 people were examined, including 28,816 people aged 1–9 years and 27,099 aged ≥15 years, with an average response rate in these age groups amongst residents of selected households of 99% and 81%, respectively. There were 547 cases of TF in children, 3 cases of TT from pre-GSM4 surveys and 3 cases of TT from post-GSM4 surveys. The prevalence of TF was ≥5% in one EU, Gashoho and Gasorwe, at baseline (Figure 1), but fell below that threshold at subsequent impact and surveillance surveys (supplementary Table S1). All EUs had an age- and gender-adjusted prevalence of TT or trichiasis unknown to the health system below the 0.2% threshold (supplementary Table S1). WASH assessments showed that, on average, 83% of households had improved drinking water sources, 49% had a drinking water source within 30 minutes, and 8% of households had improved latrines (Table 2). Washing water and drinking water, both the source and the time required to collect it, were correlated to such an extent (Pearson correlation coefficient >0.9) that it was not possible to include both variables in the regression models. Drinking water indicators were kept as proxies for the washing water equivalents.

**Table 2.** Summary of water, sanitation and hygiene access, Burundi trachoma prevalence surveys, 2018–2021.

Region	District	Survey type*	No. Households with an improved drinking water source	No. Households with drinking water source within 30 minutes	No. Households with improved latrines	% of households surveyed with an improved source of drinking water	% of households surveyed with a source of drinking water within 30 minutes	% of households surveyed with improved latrines
Kirundo	Busoni	baseline v1	550	243	49	80	35	7
Ruyigi	Butezi & Butaganzwa	baseline v1	465	432	8	67	63	1
Muyinga	Gasorwe & Gashoho	baseline v1	678	324	67	99	47	10
Muyinga	Gasorwe & Gashoho	surveillance v2	534	260	64	77	38	9
Muyinga	Gasorwe & Gashoho	impact v2	569	269	56	82	39	8
Rutana	Gitanga & Gihofi & Giharo & Bukemba	baseline v1	504	450	103	73	65	15
Karuzi	Gitaramuka & Buhiga & Bugenyuzi	surveillance v2	583	381	58	84	55	8
Muyinga	Giteranyi & Butihinda	baseline v1	638	66	77	93	10	11
Cankuzo	Kigamba & Cendajuru & Cankuzo	baseline v2	616	285	0	90	41	0
Kirundo	Kirundo & Bugabira	baseline v1	372	208	30	54	30	4
Makamba	Makamba & Kibago & Kayogoro	baseline v2	533	284	125	77	41	18
Kayanza	Muhanga & Gatara & Gahombo	baseline v1	656	412	73	95	60	11
Kirundo	Munkenke & Gitobe & Bwambarangwe	baseline v2	619	338	51	90	49	7
Gitega	Mutaho & Bugendana	baseline v1	584	477	22	85	69	3
Muyinga	Mwakiro & Muyinga & Buhinvuza	surveillance v2	627	135	69	91	20	10
Ruyigi	Nyabitsinda & Kinyinya & Gisuru	baseline v1	533	471	36	77	68	5
Kayanza	Rango & Musema & Matongo & Butaganzwal	baseline v1	652	359	36	95	52	5
Rutana	Rutana & Musongati & Mpinga-Kayove	surveillance v2	488	287	54	71	42	8
Rutana	Rutana & Musongati & Mpinga-Kayove	impact v1	539	545	38	78	79	6
Karuzi	Shombo & Nyabikere & Mutumba & Gihogazi	surveillance v2	654	299	51	95	43	7
Ngozi	Tangara & Marangara & Kiremba	baseline v1	595	380	37	86	55	5
Makamba	Vugizo & Nyanza Lac & Mabanda	baseline v2	653	555	154	95	81	22

Note: *WASH options and categories were updated in 2019 to align with the latest WHO/UNICEF Joint Monitoring Program (JMP) Core Questions for Households^33^.

### Analysis of associations

**Figure 2.**
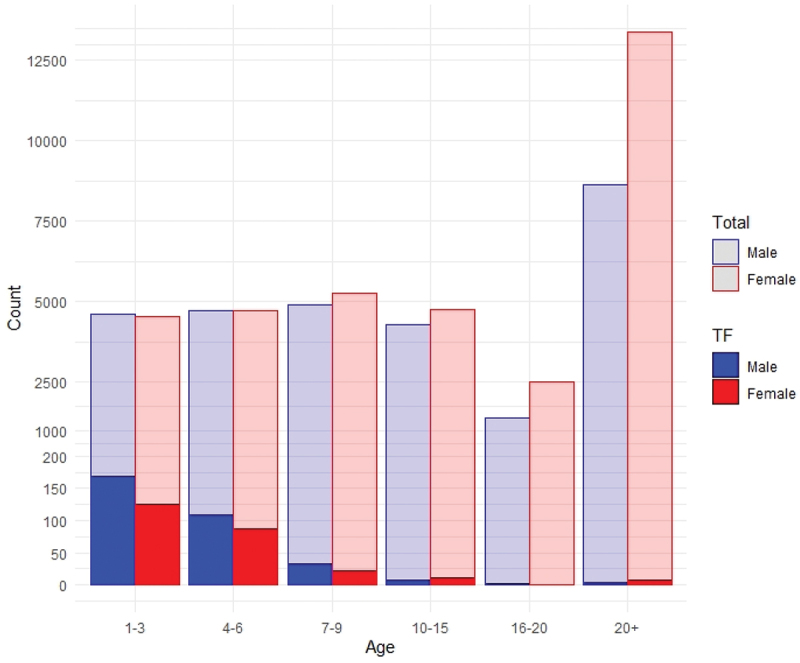
Number of people examined and number of trachomatous inflammation – follicular (TF) cases, by age group and gender, Burundi trachoma prevalence surveys, 2018–2021.

There were too few cases of TT or trichiasis (*n* = 6) to perform an association analysis. Mixed-effect models of TF showed that household was the most appropriate random effect. Regression analyses showed strong evidence of an association of TF with age and gender, with younger males more likely to have this sign (Figure 2). There was no evidence of an association between WASH variables and TF (Table 3), therefore only univariable analyses were conducted.

**Table 3.** Association analysis of water, sanitation and hygiene variables with trachomatous inflammation – follicular, Burundi trachoma prevalence surveys, 2018–2021. * indicates the reference level to which all other levels within the group were compared.

Clinical sign	Level	Univariable odds ratio (95% confidence interval)	Univariable P-value	Multivariable odds ratio (95% confidence interval)	Multivariable P-value
Age (years)	1–3	365.7 (174.4–767.8)	<0.001	499.1 (217.9–1143.0)	<0.001
	4–6	128.2 (61.6–266.5)	<0.001	162.9 (71.8–369.6)	<0.001
	7–9	10.1 (4.7–21.8)	<0.001	13.9 (5.9–32.6)	<0.001
	10–15	2.9 (1.1–7.4)	0.02	4.3 (1.6–11.4)	0.004
	16–19	0.0 (0.0 – Inf)	0.99	0.0 (0.0 – Inf)	0.78
	20+*	-	-	-	-
Gender	Female	0.4 (0.3–0.5)	<0.001		
	Male*	-	-	-	-
Ownership of latrines	Private	1.2 (0.3–5.6)	0.81	-	-
	Open	1.6 (0.0–167.3)	0.84	-	-
	Shared*	-	-	-	-
Drinking water	Improved*	-	-	-	-
	Unimproved	0.7 (0.1–7.6)	0.74	-	-
	Surface	1.0 (0.1–9.1)	0.98	-	-
Distance to drinking water	≤30 minutes	0.3 (0.00–1.9)	0.2	-	-
	More than 30 minutes*	-	-	-	-
Number of children under 10 per household	1*	-	-	-	-
	2–3	1.5 (0.4–6.6)	0.63	-	-
	4–5	1.1 (0.1–9.3)	0.93	-	-
	6+	0.8 (0.0–921.4)	0.97	-	-
Handwashing station within 15 meters	Yes	0.9 (0.1–10.2)	0.93	-	-
	No*	-	-	-	-

## Discussion

There are three criteria for trachoma elimination : a prevalence of TT unknown to the health system below 0.2%, a prevalence of TF below 5%, and evidence that the health system is able to identify and manage incident TT cases.^29^ These surveys collectively show that Burundi has a low prevalence of trachoma and fulfils the prevalence criteria for claiming trachoma elimination status. Following antibiotic MDA using azithromycin and tetracycline in the three districts with TF prevalence ≥10% (Buhiga, Nyabikere, and Muyinga) 2011 and, in 2012, in the district of Rutana, only one EU, Gashoho and Gasorwe, exceeded the active trachoma threshold indicative of a public health problem, with a TF prevalence in 1–9-year-olds at baseline of 7.2%. Following MDA, subsequent impact and surveillance survey data showed a substantial reduction in TF prevalence over time in this EU, with estimates of 3.5% and 0.3% respectively. When comparing the prevalences of EUs studied in 2009–2010^17^ with the results of the baseline surveys in the present series, it is reasonable to conclude that TF prevalence in general is declining in Burundi, as all EUs had lower TF estimates than the estimates made in 2009–2010, regardless of whether or not they received antibiotic MDA. Burundi has achieved above 80% coverage in all MDA rounds, and behavioural change has been encouraged through hygiene awareness campaigns promoted through schools, media and other communication channels. These actions, alongside improved sanitation and water supply services, may have contributed to the reduction in TF prevalence nationwide. Our estimates of TT and trichiasis suggest that the blinding stage of trachoma is not a public health problem in Burundi. This was also a conclusion of the 2007 trachoma surveys, in which only three cases of trichiasis were found in a sample of 1481 adult women.^17^ Although the 2007 survey was not sufficiently powered to precisely estimate the prevalence of TT, which is relatively rare in comparison to the more common TF, the combined data from all surveys conducted between 2007 and 2021 suggest that TT is likely to be below the 0.2% threshold throughout the country. Only one case of trichiasis was detected during the 2018 surveys and one further case was identified during the 2019 MDA. Community health workers across the country continue to search for TT cases, with any TT found to be operated on by ophthalmologists.

The association analysis results presented here suggested that male children aged <3 years were most likely to have TF, indicating that while TF prevalence is below the WHO threshold, there may still be ongoing transmission of ocular Ct. WHO guidance states that in order to gain elimination status, a country must prepare and submit a dossier containing evidence of the achievement of elimination targets with impact survey data, and follow-up surveillance survey data demonstrating sustained below-threshold levels of TF prevalence.^29^ An additional requirement is to describe national post-validation surveillance plans, including the provision of TT surgical intervention. Young children living in areas with limited WASH access have been shown to be more likely to have active trachoma,^30–32^ and one aim of post-validation surveillance is to ensure that continued trachoma interventions are targeted to these areas, where needed, in order to maintain prevalence below the 5% threshold in all EUs.

In this dataset, none of the WASH variables were associated with TF in the univariable models and were therefore not included in a multivariable model. Previous trachoma studies have shown that access to clean water and improved latrines is inversely correlated with the prevalence or likelihood of having active trachoma.^31,32^ It is possible that the number of TF cases was too low to observe this relationship if it was present in this population. Latrine ownership was also low in all EUs which would have made it more difficult (from a statistical perspective) to show a protective association with TF. Further WASH investment, specifically the F and E aspects of the SAFE strategy, are recommended in order to improve access to clean washing water and improved latrines.

A limitation of note in this study is the change in survey type between the 12 surveys conducted in 2018 using the pre-GSM4, and the 10 surveys conducted after 2019 using the post-GSM4 methodology. Changes made to the definition of TT, as previously described, mean direct comparisons of TT prevalence estimates between pre-GSM4 baseline and post-GSM4 impact and/or surveillance surveys are not possible. The pre-GSM4 surveys did not record which eyelid exhibited signs of trichiasis in each individual, and therefore the questions regarding past surgery, which determine whether a person’s trichiasis was “unknown to the health system”, were not separated by upper and lower eyelid, and so it was not possible to determine an equivalent measure as that recorded in post-GSM4 surveys. Both TT and trichiasis estimates are well below the 0.2% threshold for all surveys however, so this limitation should not affect Burundi’s claim to trachoma elimination.

Burundi has demonstrated the prevalence levels necessary for trachoma elimination status. The data from this study show that with continued effort and the maintenance of existing management plans, trachoma elimination in Burundi is within reach.
